# Molecular Analysis of UV-C Induced Resveratrol Accumulation in *Polygonum cuspidatum* Leaves

**DOI:** 10.3390/ijms20246185

**Published:** 2019-12-07

**Authors:** Zhongyu Liu, Junxiong Xu, Xiang Wu, Yanyan Wang, Yanli Lin, Duanyang Wu, Hongjie Zhang, Jianbing Qin

**Affiliations:** College of Life Science, Yangtze University, Jingzhou 434025, China; yzrs91@163.com (Z.L.); 2017714456@yangtzeu.edu.cn (J.X.); xufyz20016@126.com (X.W.); llqww17@126.com (Y.W.); linyanli1998@foxmail.com (Y.L.); kdysuperme@163.com (D.W.); zhk18672558067@163.com (H.Z.)

**Keywords:** regulation, RNA-seq, abiotic stress, biosynthesis pathway, chalcones, stilbenes

## Abstract

Resveratrol is one of the most studied plant secondary metabolites owing to its numerous health benefits. It is accumulated in some plants following biotic and abiotic stress pressures, including UV-C irradiation. *Polygonum cuspidatum* represents the major natural source of concentrated resveratrol but the underlying mechanisms as well as the effects of UV-C irradiation on resveratrol content have not yet been documented. Herein, we found that UV-C irradiation significantly increased by 2.6-fold and 1.6-fold the resveratrol content in irradiated leaf samples followed by a dark incubation for 6 h and 12 h, respectively, compared to the untreated samples. De novo transcriptome sequencing and assembly resulted into 165,013 unigenes with 98 unigenes mapped to the resveratrol biosynthetic pathway. Differential expression analysis showed that *P.* *cuspidatum* strongly induced the genes directly involved in the resveratrol synthesis, including phenylalanine ammonia-lyase, cinnamic acid 4-hydroxylase, 4-coumarate-CoA ligase and stilbene synthase (*STS*) genes, while strongly decreased the chalcone synthase (*CHS*) genes after exposure to UV-C. Since CHS and STS share the same substrate, *P. cuspidatum* tends to preferentially divert the substrate to the resveratrol synthesis pathway under UV-C treatment. We identified several members of the MYB, bHLH and ERF families as potential regulators of the resveratrol biosynthesis genes.

## 1. Introduction

*Polygonum cuspidatum* Sieb. et Zucc. is a member of the buckwheat family (Polygonaceae). It is a tall and resilient herbaceous perennial with woody rhizomes [1], native to East Asia in countries such as Korea, Japan and China. Although this plant has been recognized as an invasive species in Europe and North America [2,3], *P. cuspidatum* has an extraordinary value in phytotherapy. In China, the roots of *P. cuspidatum* have been long employed in traditional medicine to combat cough, hepatitis, infection, arthralgia, tumors, bronchitis, jaundice, bleeding, amenorrhea and hypertension [4,5,6]. Analytic investigations of the major health-promoting molecules of *P. cuspidatum* roots have revealed the presence of several lead compounds belonging to the group of polyphenols [7,8]. Distinctly, one of the most important compounds in *P*. *cuspidatum* roots, which has drawn growing interest on this species, is resveratrol, a molecule with proven anti-inflammatory and antioxidant activity [9,10].

The “French paradox”, a curious observation referring to the low level of coronary heart disease in France despite high intake of dietary cholesterol and saturated fat [11], has been linked to the high consumption of red wine containing resveratrol. Resveratrol (3,5,40-trihydroxy-trans-stilbene) is a naturally occurring stilbene metabolite found in grapes, berries, nuts and other plants such as *P. cuspidatum*. Over the past decades, extensive researches have been carried out on the physiological functions of resveratrol in human and it has been suggested that resveratrol is a potential remedy for a range of diseases, including heart disease, diabetes, cancer and Alzheimer’s disease [12]. The compound was first discovered in *P. cuspidatum* [13], which is the most important concentrated source of free or glycosylated resveratrol. Therefore, it is expected that this species should be a model plant to study resveratrol biosynthesis and engineering in plant. Surprisingly, very limited advances in these fields have been made so far in *P. cuspidatum* [14], contrasting with the extensive knowledge generated in grape (reviewed by Hasan and Bae, [15]). In fact, the lack of genomic resources for *P*. *cuspidatum* hinders genetic discoveries. Particularly, the key genes and molecular mechanisms underlying the striking accumulation of resveratrol in this species are still unknown. On the opposite, early genome sequencing of grape [16] has triggered much research on the biosynthesis of stilbenes. The biosynthetic pathway of resveratrol has been well characterized and involves four key enzymes, including phenylalanine ammonia lyase (PAL), cinnamic acid 4-hydroxylase (C4H), 4-coumarate: CoA ligase (4CL) and stilbene synthase (STS) [17]. p-coumaroyl-CoA is a product of PAL, which is abundant in plants and used as a precursor for the synthesis of both resveratrol and chalcone. Therefore, in stilbene-synthesizing plants, STS competes with chalcone synthase (CHS) for the synthesis of resveratrol [18]. 

Resveratrol is basically a defense compound (phytoalexin) in plants but it is produced in very small amounts [19]. Therefore, how to induce a strong accumulation of resveratrol in plant organs in order to satisfy the increasing global demand of resveratrol has become one of the hot topics in secondary metabolite research. Manipulation of different synthetic enzymes and the identification of their regulator genes such as *MYB14*, *MYB15* and *WRKY8* [20,21] provide currently prospects to increase resveratrol production in planta. Moreover, it has been demonstrated that biotic and abiotic factors, including fungal attack, UV-C irradiation, jasmonic acid, salicylic acid, H_2_O_2_ and AlCl_3_, can induce the accumulation of resveratrol in grape [22,23,24,25,26,27,28]. 

Hao et al. [14] previously identified 54 unigenes predicted to participate in the resveratrol biosynthesis pathway in *P. cuspidatum* roots. However, the mechanism leading to the high resveratrol accumulation in this species and the responses of these genes to abiotic factors such as UV-C irradiation remain open to study. Here, we investigated the effect of UV-C irradiation on leaf resveratrol content in *P. cuspidatum* and further de novo sequenced the transcriptome, offering a novel insight into the UV-C induced biosynthesis of resveratrol in plants.

## 2. Results

### 2.1. Effect of UV-C Irradiation on Leaf Resveratrol Content in Polygonum cuspidatum

**Figure 1:**
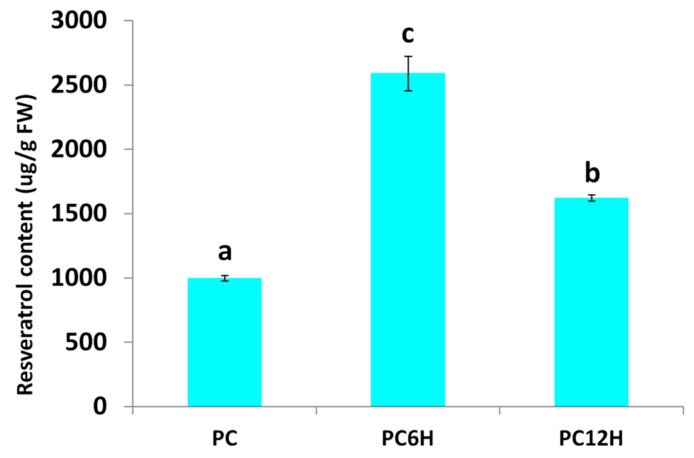
Effect of UV-C irradiation and incubation times on the resveratrol contents in *Polygonum*.

*Polygonum cuspidatum* leaves were treated with UV-C light for 10 min and then incubated in the dark for 6 h (PC6H) and 12 h (PC12H). We quantified the leaf resveratrol accumulation in UV-C treated leaves and untreated leaves (PC). As shown in Figure 1, we observed a significant difference in the resveratrol contents of UV-C treated leaves as compared to untreated leaves (*p* < 0.001) and between the different incubation times of treated leaves (*p* < 0.001). UV-C significantly increased the leaf resveratrol contents and 6 h incubation time after UV-C treatment showed the highest accumulation of resveratrol in the leaves of *P. cuspidatum*.

### 2.2. De Novo Transcriptome Sequencing and Unigene Assembly

In order to elucidate the molecular mechanism underlying the UV-C induced resveratrol accumulation in *Polygonum cuspidatum* leaves, we synthesized nine cDNA libraries from leaf samples of PC12H, PC6H, PC and generated de novo RNA-sequencing data.

**Figure 2:**
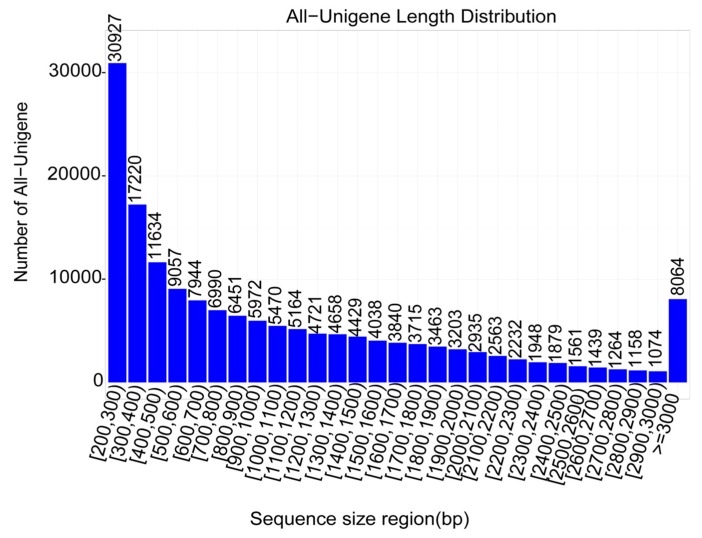
Unigene length distribution. The numbers above the bar represent the number of unigenes with a sequence length comprised in the classes of size displayed in the x-axis.

A total of 73.63 Gb raw read data was generated. After cleaning, 98.7% of the reads were kept as clean reads with 93.5% of bases scoring Q30 and above (Table 1). The assembly was performed using the Trinity software and a total of 165,013 unigenes were obtained with N50 length about 1744 bp (Table 2). The unigene lengths ranged from 200 to 17,269 bp with a significant number of genes having length of 200–300 bp (Figure 2).

**Figure 3:**
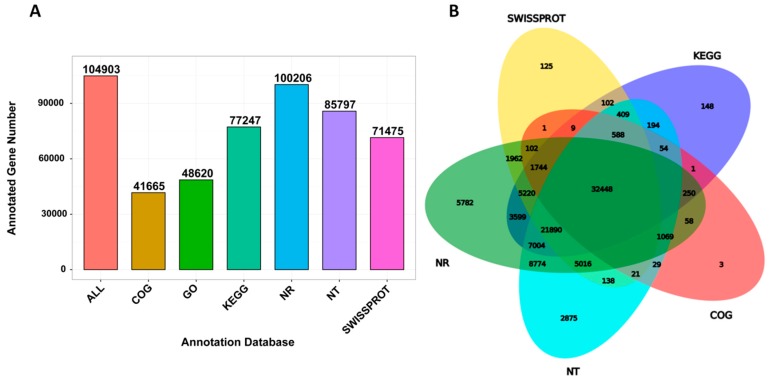
Unigene functional annotation in *P. cuspidatum*. (**A**) The annotated gene number in different databases and (**B**) Venn diagram showing the number of shared and unique annotated genes in the different databases.

We performed the functional annotation of the unigenes in various databases, including NR, NT, Swiss-Prot, Kyoto Encyclopedia of Genes and Genomes (KEGG), Clusters of Orthologous Groups (COG) and Gene Ontology (GO), which resulted in 104,903 annotated unigenes (Figure 3A) with 32,448 unigenes successfully annotated based on all the five databases (Figure 3B). Overall, these unigenes contributed to various GO terms of which the most represented ones were binding, catalytic activity and transporter activity (molecular function). They were mainly located in the cell, cell part and membrane (cell component) and involved in the cellular process, metabolic process, etc. (biological process; Figure S1). We further analyzed the genes encoding for transcription factors within the annotated unigenes. As shown in Table 3, we identified 5526 TFs classified into 58 different TF families with bHLH, MYB, bZIP and ERF as the most abundant TFs. In addition, we searched for all genes belonging to the resveratrol biosynthetic pathway and identified 98 unigenes including, 26 phenylalanine ammonia-lyase (PAL), 15 cinnamic acid 4-hydroxylase (C4H), 20 4-coumarate-CoA ligase (4CL), 4 stilbene synthase (STS) and 33 chalcone synthase (CHS; Table S1).

**Figure 4:**
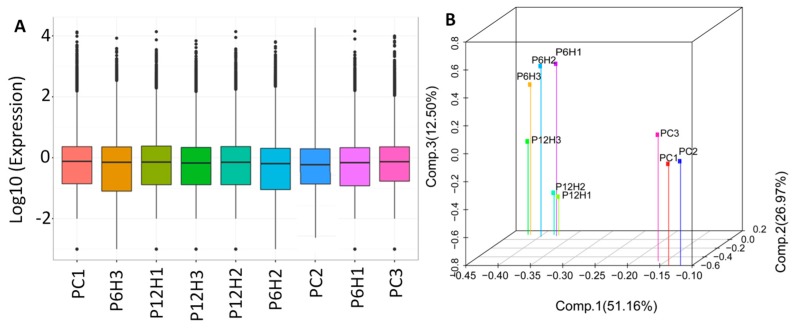
Overview of the transcriptome sequencing in *P. cuspidatum* leaves. (**A**) Gene expression profiles in the nine libraries. PC, PC6H and PC12H represent samples collected before, 6 h and 12 h after UV-C treatment, respectively and (**B**) 3D principal component analysis showing clustering pattern among leaf samples based on global gene expression profiles.

Next, the clean reads data were mapped to the assembled unigene libraries and the statistics of mapping results are presented in Table S2. A total of 157,665 genes were expressed with the number of fragments per kilobase of exon per million fragments mapped (FPKM) values raging from 0.02 to 18,562.92 (Figure 4A). Principal component analysis (PCA) of the samples based on FPKM showed that all the biological replicates clustered together, suggesting a high reliability of our RNA-sequencing data (Figure 4B). Furthermore, the PCA clearly distinguished the control and the UV-C treated leaf samples, indicating that a large number of genes were differentially expressed after exposure to UV-C irradiation.

### 2.3. Differential Expressed Genes (DEG) between Control and UV-C Treated Leaf Samples

**Figure 5:**
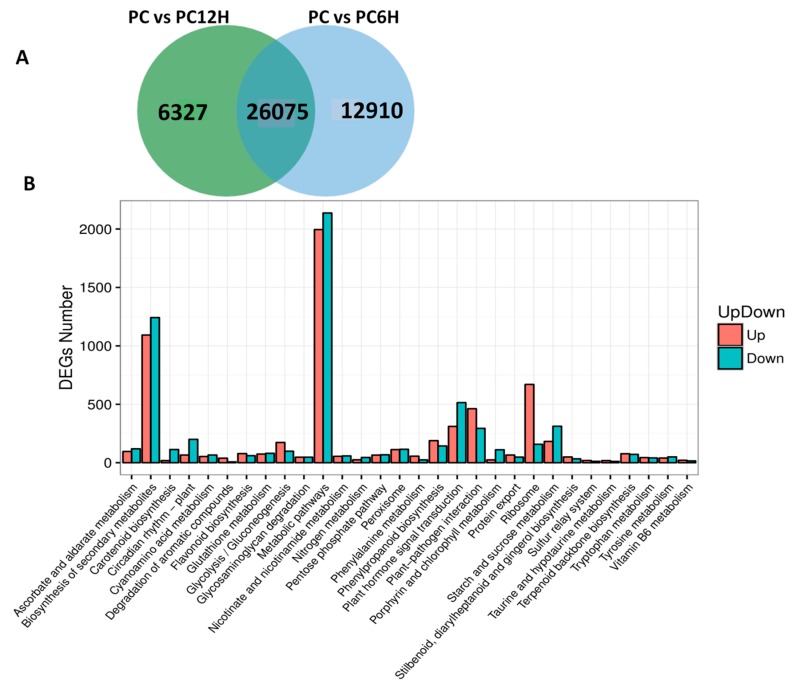
Differentially expressed genes (DEG) between the UV-C treated samples and the untreated control. (**A**) Venn diagram showing the unique and conserved DEGs between PC vs. PC6H and PC vs. PC12H; (**B**) Kyoto Encyclopedia of Genes and Genomes (KEGG) enrichment analysis of all the identified DEGs. PC, PC6H and PC12H represent samples collected before, 6 h and 12 h after UV-C treatment, respectively.

We compared the gene expression levels between control samples (PC) to UV-C treated samples PC6H and PC12H with the aim to detect the genes affected by the UV-C treatment. Concerning PC vs. PC6H, we identified 38,985 differentially expressed genes (DEGs) with 17,859 and 21,126 up- and down-regulated, respectively. A slightly lower number of DEGs (32,312) was found for PC vs. PC12H, including 14,416 and 17,896 up and down-regulated genes, respectively. Cross-comparison of the two sets of DEGs showed that in total 45,222 genes were affected by the UV-C treatment and 26,075 DEGs were constantly conserved after 6 h and 12 h post UV-C irradiation (Figure 5A). These genes represent the key genes involved in the metabolic changes in response to UV-C treatment. To get insight into the biological pathways contributed by these DEGs, we performed KEGG enrichment analysis. The results indicated that the DEGs play various roles but were mainly involved in metabolic pathways and biosynthesis of secondary metabolites. In addition, ribosome, plant hormone transduction and phenylpropanoid biosynthesis were the other enriched pathways contributed by these DEGs (Figure 5B).

Gene expression levels are modulated by regulators such as transcription factors [29]. We extended the analysis to detect the major transcription factor families involved in the response to UV-C exposure in the *Polygonum cuspidatum* leaf. Surprisingly, nearly half (2461) of the total annotated TF genes (5526) in *P. cuspidatum* was found differentially expressed and included 2132 and 1895 TFs DEGs detected at 6 h and 12 h, respectively. The majority of these genes were MYB, bHLH and ERF family members (Table S3).

### 2.4. Mapping of DEGs Related to the Resveratrol Biosynthetic Pathway

Deamination of phenylalanine ammonia by PAL is the first step in the resveratrol biosynthesis pathway. Then, the conversions of cinnamic acid into p-coumaric acid and subsequently into 4-coumarate-CoA are catalyzed by C4H and 4CL, respectively. The last step in the pathway consists of the conversion of one 4-coumarate-CoA and three malonyl-CoA units into resveratrol or naringenin chalcone by STS or CHS, respectively. Later, resveratrol is converted into pterostilbene by resveratrol O-methyltransferase (ROMT). We searched within the DEGs all genes encoding the key enzymes involved in the resveratrol biosynthesis pathway and successfully identified 37 related DEGs, including 36 and 34 at 6 h and 12 h post UV-C exposure, respectively. These genes included 10 PAL, eight *C4H*, nine *4CL*, two *STS*, six *CHS* and two *ROMT*.

**Figure 6:**
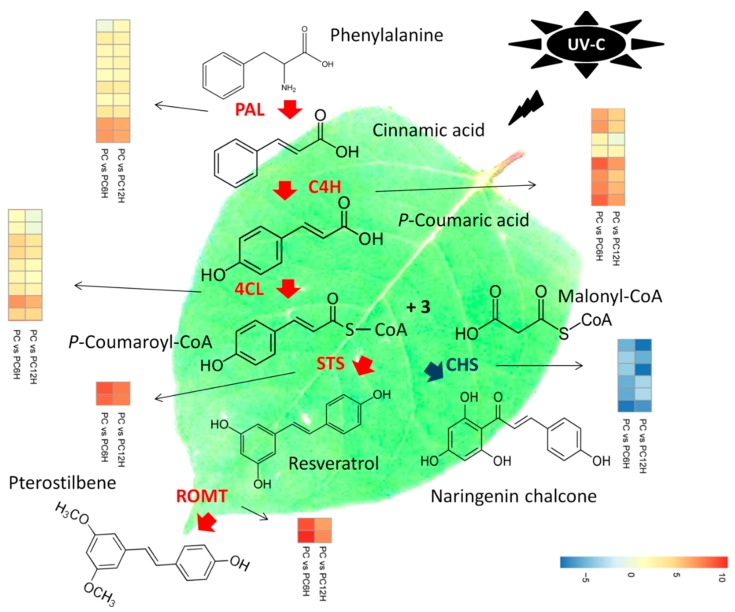
Proposed model of the molecular mechanism leading to the high accumulation of resveratrol after treatment with UV-C in *Polygonum cuspidatum* leaf. Deamination of phenylalanine ammonia by phenylalanine ammonia-lyase (PAL) is the first step in the resveratrol biosynthesis pathway. Then, the conversions of cinnamic acid into *p*-coumaric acid and subsequently into 4-coumarate-CoA are catalyzed by cinnamic acid 4-hydroxylase (C4H) and 4-coumarate-CoA ligase (4CL), respectively. The last step in the pathway consists of the conversion of one 4-coumarate-CoA and three malonyl-CoA units into resveratrol or naringenin chalcone by stilbene synthase (STS) or chalcone synthase (CHS), respectively. Later, resveratrol is converted into pterostilbene by resveratrol O-methyltransferase (ROMT). PAL, C4H, 4CL and STS were found strongly up-regulated in PC6H and PC12H as compared to PC, while CHS displayed the opposite trend. STS and CHS share the same substrate. *P. cuspidatum* tends to prioritize resveratrol accumulation by diverting the substrate “one 4-coumarate-CoA and three malonyl-CoA units” to the resveratrol synthesis pathway over the naringenin chalcone synthesis pathway through up-regulation of STS genes and down-regulation of CHS genes in response to UV-C exposure. High induction of ROMT also suggests that pterostilbenes may also be accumulated. PC, PC6H and PC12H represent samples collected before, 6 h and 12 h after UV-C treatment, respectively.

We mapped the 37 DEGs related to the resveratrol biosynthesis along with their gene expression fold change in order to understand the effect of UV-C radiation on resveratrol biosynthesis (Figure 6). Interestingly, we observed that all the 29 genes directly involved in the resveratrol biosynthesis (*PAL, C4H, 4CL* and *STS*) were significantly up-regulated after exposure to UV-C. Meanwhile, the six *CHS* genes were found strongly down-regulated. Concerning the DEGs encoding ROMT, all of the two genes were found sharply induced by UV-C treatment in *Polygonum cuspidatum* leaf (Figure 6). Globally, it appeared that the regulation of these 37 key genes was more intense after 6 h incubation of the leaves in the dark post UV-C irradiation as compared to 12 h.

### 2.5. qRT-PCR Validation of Candidate Genes

**Figure 7:**
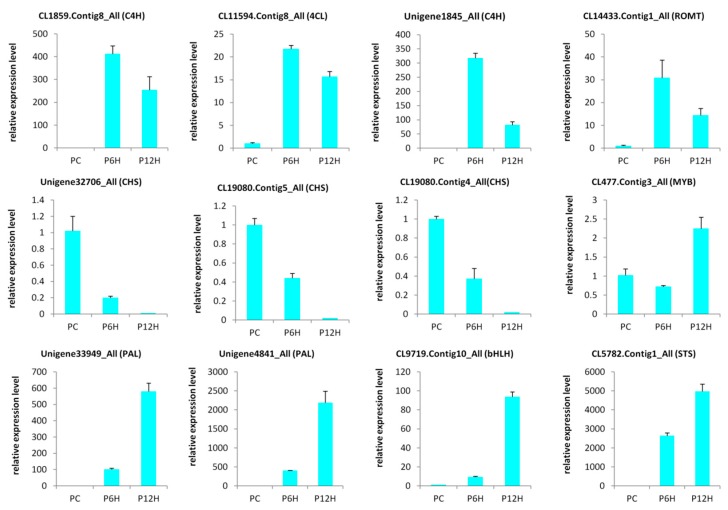
Quantitative real time PCR validation of selected candidate genes. The error bar represents the SD of three biological replicates. The *Actin* gene was used as the internal reference gene for normalization; PC, PC6H and PC12H represent samples collected before, 6 h and 12 h after UV-C treatment, respectively.

In order to validate the results obtained through the RNA-seq, we selected 12 candidate DEGs from the enzymes involved in the resveratrol biosynthesis and some highly regulated transcription factors genes and conducted a qRT-PCR analysis (Table S4). As shown in the Figure 7, the expression levels of all the tested genes were significantly altered after treatment with UV-C and the qRT-PCR results were strongly correlated with the RNA-seq reports (R^2^ = 0.81, Figure S2). These results demonstrate the reliability of the RNA-seq data obtained in the present study.

## 3. Discussion

Resveratrol exhibits diverse beneficial properties in humans, including anti-inflammatory effects, anti-tumor activities and anti-aging effects, which have drawn extensive studies on this precious molecule [12]. As a phytoalexin, resveratrol plays important functions as an antimicrobial and antioxidant compound in plant responses to environmental stresses, such as UV irradiation and fungal infection [15]. Plants have developed various alleviation mechanisms to mitigate short wavelength UV-C radiation damaging effects, including a strong accumulation of UV-absorbing phenolic and flavonoid molecules in epidermal cells to block light penetration and anti-oxidative molecules to limit photo-oxidative damage [30,31,32]. In grape, UV-C irradiation induces a strong accumulation of resveratrol [28,33,34]. It was reported that UV-C could significantly stimulate the synthesis of resveratrol in *Vitis vinifera* × *V. amurensis*, mainly in the form of trans-resveratrol [35]. Similarly, UV-C treatments increased resveratrol synthesis in *Gnetum parvifolium* [36]. These reports suggest that the UV-C induction of resveratrol is quite conserved in stilbenoid-synthesizing plants. Although, *P. cuspidatum* represents the highest natural source of resveratrol known to date, no information is available regarding the effect of abiotic stress such as UV-C treatment on the level of resveratrol. Herein, we studied the effect of UV-C irradiation on the resveratrol metabolism in *P. cuspidatum* mature leaves. As expected, we found that *P. cuspidatum* leaf contained a very high level of resveratrol (1000 µg/g FW) and UV-C could significantly induce the resveratrol accumulation (Figure 1), indicating that UV-C irradiation represented a good prospect for increasing resveratrol content in *P. cuspidatum*. Different UV irradiation treatments resulted in various levels of resveratrol, therefore an optimized UV treatment protocol is important for a significant induction of resveratrol. In grape, irradiation strength ranging from 30 to 510 W up to 1 min, followed by incubation in 2–4 days, resulted in 10.8-fold higher accumulation of resveratrol than that observed in the untreated control [37,38]. Cantos et al. [39] also showed that a distance of 40 cm, irradiation time of 30 s, source power of 500 W and storage time of 3 days generated the highest resveratrol accumulation. In this study, we incubated the UV-C irradiated leaves for 6 and 12 h. We observed an increase of 2.6-fold and 1.6-fold of resveratrol after 6 h and 12 h incubation times, respectively, suggesting that the effect of the UV-C treatment diminished after 6 h. Although the induction levels were not high as compared to studies in grape [37,38], based on our results and pending more optimization of the UV-C treatment protocol, we might recommend 10 min UV-C irradiation and 6 h incubation in the dark for obtaining a relatively high resveratrol in *P. cuspidatum* leaves.

To understand the molecular mechanism underlying the strong accumulation of resveratrol in *P. cuspidatum* after exposure to UV-C, we de novo sequenced the transcriptome and assembled the unigenes (Table 1 and Table 2; Figure 2). In total, 165,013 unigenes were identified in this study, a number that is quite the double of the unigenes number (86,418) detected by Hao et al. [14]. The high discrepancy between the detected unigene numbers in both studies could result from differences in the tissue types, the employed software for assembly and more importantly the sequencing platform. Differential gene expression (DEG) analysis showed that UV-C treatments affected nearly ¼ of the expressed genes and various cellular processes including hormones and secondary metabolism were altered (Figure 5). Our results were in perfect concordance with works by Yin et al. [40], who demonstrated that more than 100 functional subcategories were contributed by the DEGs between UV-treated grape berries and untreated samples and particularly, “response to stress” and “metabolic processes” were the most represented terms.

Previous studies have demonstrated that UV-C irradiation alters the activity levels of several structural genes participating in the resveratrol biosynthesis pathway [35,36,40,41,42]. Within the DEGs detected in this study, 37 were mapped to the resveratrol biosynthesis pathway (Figure 6). Interestingly, all the genes directly involved in the resveratrol biosynthesis (10 phenylalanine ammonia-lyase (PAL), 8 cinnamic acid 4-hydroxylase (C4H), 9 4-coumarate-CoA ligase (4CL) and 2 stilbene synthase (STS)) were significantly up-regulated in UV-C treated samples as compared to untreated samples (Figure 6). Conversely, the six chalcone synthase (CHS) DEGs were all down-regulated in samples exposed to UV-C (Figure 6). In fact, CHS and STS enzymes use the same substrate (one 4-coumarate-CoA and three malonyl-CoA units) for the production of naringenin chalcone and resveratrol, respectively. This result indicates that after exposure to UV-C, *P. cuspidatum* diverts the substrate “one 4-coumarate-CoA and three malonyl-CoA units” to the resveratrol synthesis pathway over the naringenin chalcone synthesis pathway by up-regulating STS and down-regulating CHS. The conclusions of several authors, including Xi et al. [41], Suzuki et al. [42] and Yin et al. [40] support well our findings as they demonstrated that since STS and CHS share the same substrate, there may be a competitive and/or inhibitory relationship between them in response to UV-C exposure, which may in turn play a vital role in resveratrol accumulation in grape berries.

The tyrosine ammonia-lyase (TAL) enzyme can also utilize L-tyrosine as a substrate to produce p-coumaric acid, which subsequently is converted into resveratrol by STS [43]. In this study, we did not find any *TAL* gene among the DEGs, indicating that the high accumulation of resveratrol after UV-C treatments was essentially due to the strong conversion of phenylalanine and cinnamic acid through PAL and C4H, respectively (Figure 6).

Trans-resveratrol is highly instable upon exposure to light and oxygen or under harsh PH, leading to the reduction its bioavailability and bioactivity [44]. Other natural stilbenes derived from resveratrol such as pterostilbene and pinostilbene, display higher oral bioavailability and bioactivity, therefore, efforts are ongoing to develop strategies to obtain these bioactive resveratrol derivatives in abundance. An efficient technique to achieve this goal is the manipulation of resveratrol O-methyltransferase (ROMT), which is the enzyme that converts resveratrol into pterostilbenes [27,45,46]. Here, we observed that 2 ROMT genes were significantly up-regulated in UV-C treated *P. cuspidatum* leaf samples (Figure 6), suggesting that UV-C treatments not only increase the trans-resveratrol levels but may also increase the methylated derivatives of resveratrol. Comparing 6 h and 12 h incubation times with respect to the expression levels of resveratrol biosynthesis related DEGs, we found that gene induction/repression was more vigorous at 6 h than 12 h, which correlates with the higher resveratrol content observed at this time point through resveratrol quantification.

Transcription factors (TF) such as MYB and bHLH were reported to regulate the expression levels of several genes involved in the phenylpropanoid pathways [47,48]. Following UV-C treatment, the gene Myb14 has been shown to activate *STS* genes in grape [20,35,40,49]. In this study, we found 2461 TFs among the DEGs with members of MYB, bHLH and ERF being the most active after UV-C irradiation (Table S3) and might play crucial roles in UV-C induced responses in *P. cuspidatum*. We did not observe a clear trend in the differential expression of genes belonging to these families as many family members were either down-regulated or up-regulated after exposure to UV-C. This highlights the complex mechanisms of UV-C transcriptional regulation and renders the identification of potential master regulators of resveratrol biosynthetic genes very difficult. Gene co-expression analysis is a widely used technique to break down large transcriptome datasets into co-expressed modules in order to pinpoint key TFs modulating important structural genes [50,51,52,53,54,55,56]. For example, this approach has been used to discover WRKY TFs (WRKY24/28/29/37/41) that were co-expressed simultaneously with eight *STS* genes (*STS12/13/16/17/18/24/27/29*) in roots and leaves of *Vitis vinifera* [57]. Later on, TFs belonging to different families such as MYB, WRKY and ERF were shown to putatively contribute to STS regulation [58,59]. Therefore, we proposed further RNA-sequencing in *P. cuspidatum* using various genotypes and tissues to comprehensively undertake an integrated gene co-expression analysis in order to find out the key regulators of the resveratrol biosynthetic genes in this important medicinal plant species.

## 4. Materials and Methods 

### 4.1. Plant Materials

*Polygonum cuspidatum* Sieb. et Zucc. was used as plant material in this study. Plants were maintained in the medicinal plant garden at Yangtze University, Jingzhou, China. Healthy, mature (30-days old) leaves of similar size were detached from the shoot and were immediately immersed in water and subsequently transferred into ddH_2_O [60]. All leaves were incubated in the dark at 25 °C for 30 min. Then, the leaf abaxial surfaces were exposed for 10 min to 6 W/m^2^ UV-C irradiation provided by a UV-C lamp (Model CJ-1450, Sujie Purification, China). Samples were collected at 0 h (before UV-C treatment, PC), 6 h (PC6H) and 12 h (PC12H) after the initiation of treatment. Each treatment was performed three times and each replication consisted of six leaves. After sampling, the leaves were ground into powder in liquid nitrogen and stored at −80 °C until analysis.

### 4.2. Quantification of Resveratrol in Polygonum cuspidatum Leaves

The sample preparation, extract analysis, resveratrol identification and quantification were performed at Wuhan MetWare Biotechnology Co., Ltd, Wuhan, China. (www.metware.cn) following their standard procedures and previously described by Zhang et al. [61]. The experimental measurements were carried out in triplicate and results were presented as average of three analyses ± standard deviation. Statistical analysis of the data was done using the (www.r-project.org) using the one-way analysis of variance for testing statistical significance between different samples. Mean comparisons were done using the Tukey HSD test.

### 4.3. RNA Extraction, cDNA Library Construction and Transcriptome Sequencing

The leaf samples from PC, PC6H and PC12H were used for total RNAs extraction employing the Spin Column Plant total RNA Purification Kit according to the manufacturer’s protocol (Sangon Biotech, Shanghai, China). Purity of the extracted RNAs was assessed on 1% agarose gel followed by a NanoPhotometer spectrophotometer (IMPLEN, Westlake Village, CA, USA). RNA quantification was performed using the Qubit RNA Assay Kit in Qubit 2.0 Flurometer (Life Technologies, Carlsbad, CA, USA). Next, RNA integrity was checked by the RNA Nano 6000 Assay Kit of the Agilent Bioanalyzer 2100 system (Agilent Technologies, Santa Clara, CA, USA).

Sequencing libraries was created using NEB Next Ultra RNA Library Prep Kit following the manufacturer’s instructions. The index codes were added to each sample. Briefly, the mRNA was purified from 3 μg total RNA of each of three replicate using poly-T oligo-attached magnetic beads and then broken into short fragments to synthesize first strand cDNA. The second strand cDNA synthesis was subsequently performed using DNA Polymerase I and RNase H. PCR was carried out with Phusion High Fidelity DNA polymerase using universal PCR primers and index (X) primer. Finally, six paired-end cDNA libraries with an insert size of 150 bp were constructed for transcriptome sequencing and sequenced on HiSeqTM 2000 platform (Illumina Inc., San Diego, CA, USA).

### 4.4. Quality Check, Cleaning and de novo Assembly

The clean reads were retrieved after trimming adapter sequences, removal of low quality (containing >50% bases with a Phred quality score <10) and reads with unknown nucleotides (more than 5% ambiguous residues N) using the FastQC tool (http://www.bioinformatics.babraham.ac.uk/projects/fastqc/). The high quality reads from all the nine libraries were de novo assembled into transcripts using the Trinity software (version r20140717, [62]) with the following parameters: -min-contig-length, 100-min-glue and 3--path-sing-distance-85-min-kmer-cov3. Next, the transcripts were realigned to construct unigenes and the software TGICL, version v2.1 [63] was employed to eliminate the redundant unigenes and to get the final unigene list based on the following parameters: -l, 40, -c, 10, -v, 25, -O, ’-repeat_stringency, 0.95, -minmatch, 35 and -minscore 35’.

### 4.5. Functional Annotation of the Unigenes

The assembled unigenes were annotated by searching against various databases such as Kyoto Encyclopedia of Genes and Genomes (KEGG) [64], Gene Ontology (GO) [65], Clusters of Orthologous Groups (COG) [66], Swissprot [67], NR [68], euKaryotic Orthologous Groups (KOG) [69] and NT using BLAST version:v2.2.26 [70] with a threshold of *e*-value < 1 × 10^−5^. Based on the functional annotation results, according to the database priorities of NR, SwissProt, KEGG and COG, we selected the best comparison fragment of unigenes as the CDS for the unigenes. For unigenes, which failed to return a hit, we then used ESTScan v3.0.2 [71] to make predictions using default parameters.

### 4.6. Analysis of Transcription Factors

To identify the gene encoding transcription factors (TF), we first collected the HMM profiles of known transcription factors from various databases (PlantTFDB [72], AnimalTFDB [73], FTFD [74] and DBD [75]) and the literature, and then used the hmmsearch of the HMMER package version 3.1b2 to compare the protein sequence to the HMM of known TFs, with the *e*-value < 1 × 10^−5^. 

### 4.7. Gene Expression and Differential Expression Analysis

The clean reads were compared to the assembled unigenes using the tool Bowtie2 version 2.1.0 [76] and then the number of reads on each unigene was calculated using the RSEM version 1.2.21 [77]. The gene expression level was determined according to the fragments per kilobase of exon per million fragments mapped (FPKM).

The read count was normalized and DESeq2 tool version 1.4.5 [78] was used to detect the differential expressed genes (DEGs) between the control samples (PC) and the UV-C treated samples (PC6H and PC12H) with the fold change of >2 [79] and false discovery rate (FDR) correction set at *p* < 0.01. GO enrichment analysis was performed using the GOseq tool version 1.16.2 [80] with *p* < 0.05. KEGG pathway enrichment analysis of the DEGs was done using KOBAS2.0 [81] with FDR correction (*p* < 0.05).

### 4.8. Gene Expression Using Quantitative Real Time-PCR

The qRT-PCR was performed on RNA extracted from control and stressed leaf samples as described by Dossa et al. [82] using the *Actin* gene as the internal control. Specific primer pairs of 12 selected genes were designed using the Primer Premier 5.0 [83] (Table S4). The qRT-PCR was conducted on a Roche Lightcyler® 480 instrument using the SYBR Green Master Mix (Vazyme), according to the manufacturer’s protocol. Each reaction was performed using a 20 μL mixture containing 10 μL of 2× ChamQ SYBR qPCR Master Mix, 6 μL of nuclease-free water, 1 μL of each primer (10 mM) and 2 μL of four-fold diluted cDNA. All of the reactions were run in 96-well plates and each cDNA was analyzed in triplicate. The following cycling profile was used: 95 °C for 30 s, followed by 40 cycles of 95 °C/10 s, 60 °C/30 s. Data are presented as relative transcript level based on the 2^−∆∆Ct^ method [84].

## 5. Conclusions

In summary, we showed that UV-C treatments induced the accumulation of trans-resveratrol in *P. cuspidatum* leaves. Various UV-C treatments generated different levels of accumulation suggesting that there was room for optimization of the UV-C treatment protocol in order to yield maximum content of resveratrol. We further provided the putative genes participating in the resveratrol biosynthetic pathway and highlighted the key genes differentially expressed upon exposure to UV-C. It was evident that *P. cuspidatum* prioritized the resveratrol synthesis by strongly up-regulating the genes directly involved in this pathway and shutting down the expression of chalcone synthase genes. The results from this study provided insights into the mechanism of UV-C induced accumulation of resveratrol and probably its methylated forms in a species other than grape. It lays the foundation for further enhancement of stilbenes in *P. cuspidatum*.

## Figures and Tables

**Table 1:** Overview of the transcriptome sequencing dataset and quality check. *Polygonum cuspidatum* mature leaves. PC, PC6H and PC12H represent samples collected before, 6 h and 12 h after UV-C treatment, respectively. The letters above the bar represent significant difference between samples.

Type	Read Length	Total Clean Reads	Clean Reads Q20 (%)	Clean Reads Q30 (%)	Detected Genes
P12H1	150	55,184,328	97.81	93.76	122,534
P12H2	150	57,685,708	97.47	93.21	123,131
P12H3	150	60,727,642	97.83	93.85	124,005
P6H1	150	51,083,608	97.66	93.41	122,200
P6H2	150	53,076,416	97.64	93.36	120,570
P6H3	150	48,587,712	97.74	93.56	119,464
PC1	150	53,966,128	97.52	93.14	123,390
PC2	150	51,267,598	97.5	93.19	117,489
PC3	150	52,633,030	97.77	93.69	125,163

**Table 2:** Statistics of the unigene assembly results.

Sample	Total_Number	Min_Length	Max_length	Mean_Length	N50	N90	GC
All	165,013	200	17,269	1,102.73	1,744	489	0.4216

**Table 3:** Statistics of genes encoding for transcription factors.

TF	Count	TF	Count	TF	Count
LFY	3	M-type	5	HB-PHD	17
SAP	23	GATA	105	E2F/DP	23
WRKY	298	DBB	44	Trihelix	126
RAV	13	ZF-HD	35	ARF	137
CPP	35	LBD	50	MIKC	98
HRT-like	3	MYB	402	SRS	15
HB-other	45	LSD	14	NF-X1	8
FAR1	67	WOX	15	Nin-like	43
ERF	341	C2H2	204	MYB_related	272
Whirly	11	CAMTA	23	VOZ	18
ARR-B	57	GRAS	109	G2-like	157
HSF	90	GRF	32	NF-YA	56
NZZ/SPL	8	EIL	36	Dof	119

## Supplementary Materials

Supplementary materials can be found at https://www.mdpi.com/1422-0067/20/24/6185/s1. Figure S1 Gene ontology classification of all unigenes detected in this study; Figure S2 Correlation of the qRT-PCR expression profiles and the RNA-seq based on the selected genes. PC, PC6H and PC12H represent samples collected before, 6 h and 12 h after UV-C treatment, respectively; Table S1 List of the genes encoding enzymes involved in the resveratrol biosynthetic pathway; Table S2 Statistics of the read mapping results; Table S3 List of the differentially expressed transcription factors detected in this study; Table S4 The primer sequences of genes used for real time quantitative PCR.
