# Supplementary figures and images for: Molecular Analysis of UV-C Induced Resveratrol Accumulation in Polygonum cuspidatum Leaves
Source: Int J Mol Sci. 2019 Dec 7;20(24):6185. doi: 10.3390/ijms20246185 (PMC6940797; doi:10.3390/ijms20246185)

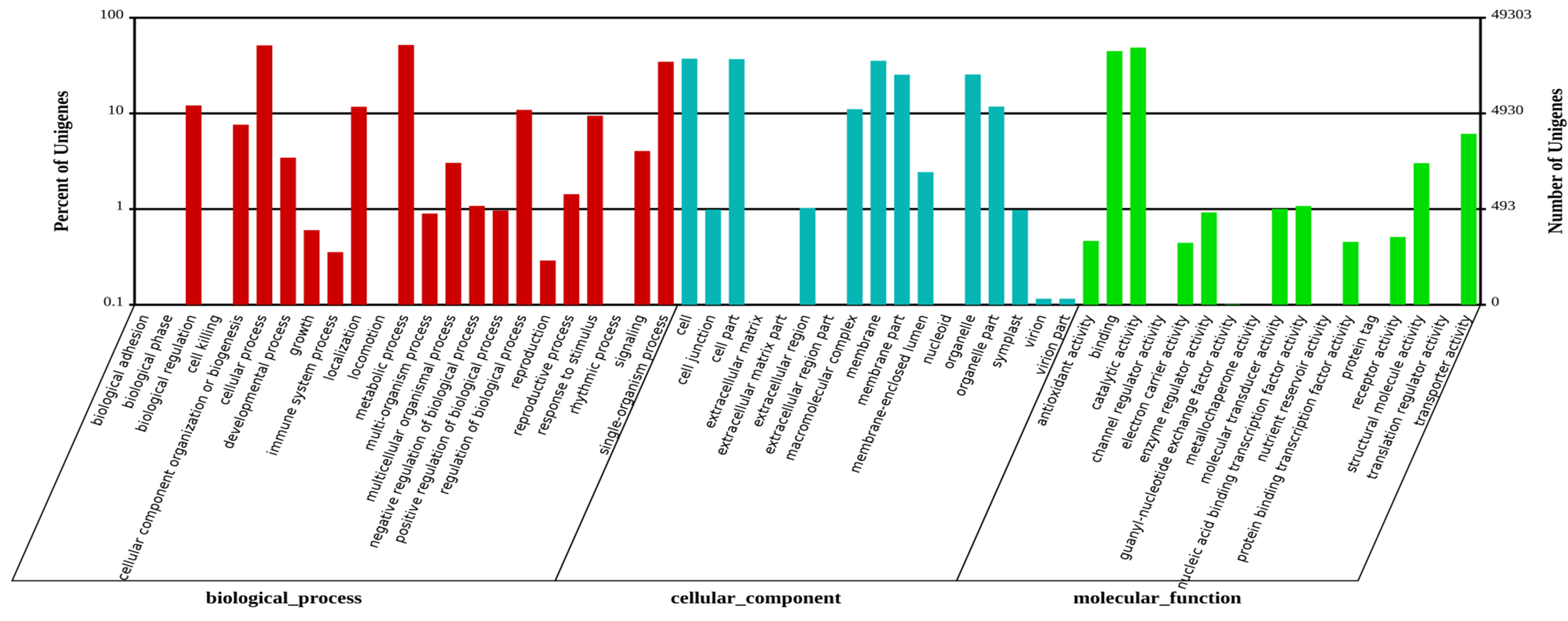

Supplement: Supplementary file 1 [file ijms-20-06185-s001.zip › ijms-652535-supplementary/Figure S1.tif]

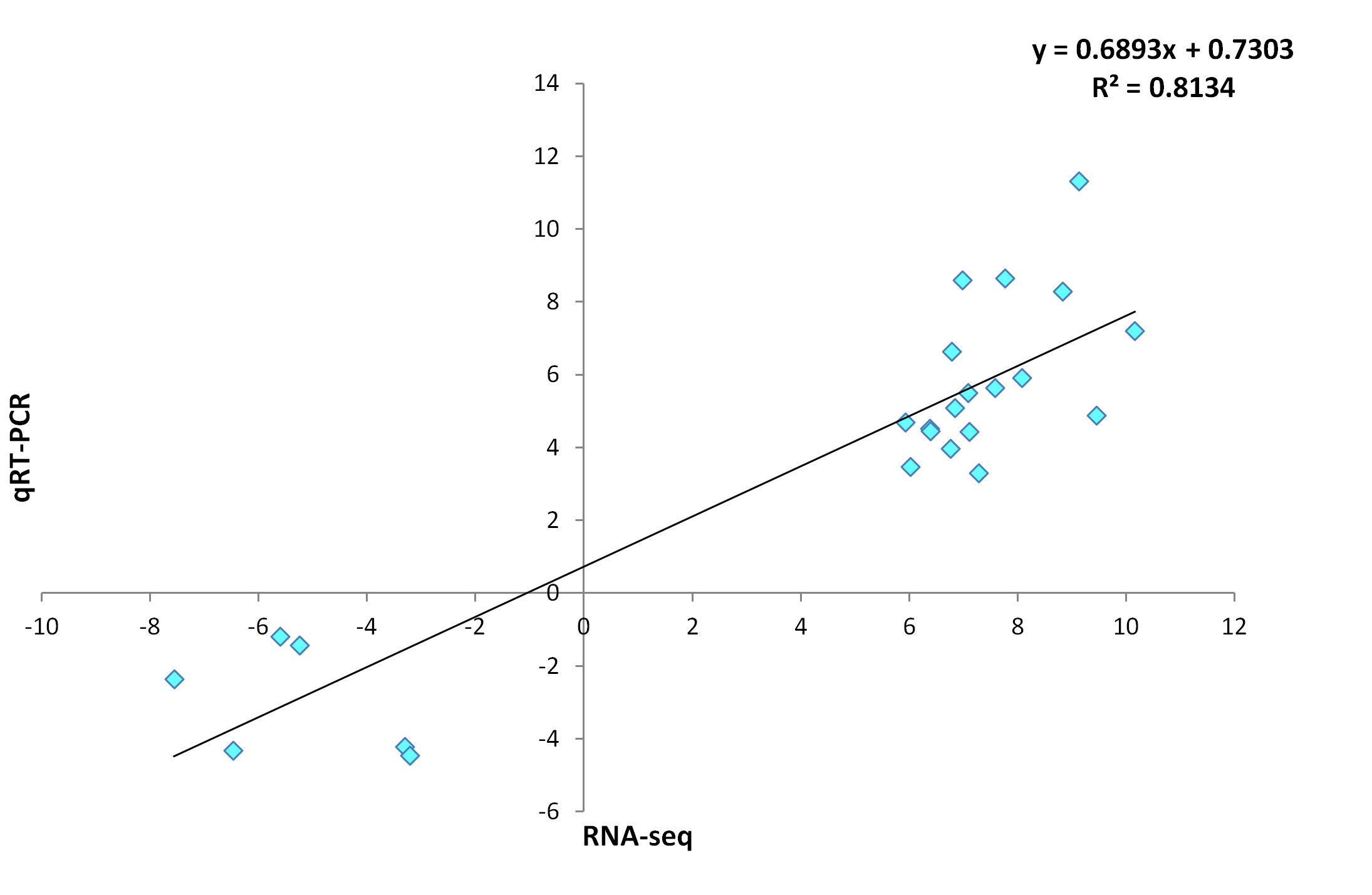

Supplement: Supplementary file 1 [file ijms-20-06185-s001.zip › ijms-652535-supplementary/Figure S2.tif]
